# Supplementary material for: Hybrid Models and Biological Model Reduction with PyDSTool
Source: PLoS Comput Biol. 2012 Aug 9;8(8):e1002628. doi: 10.1371/journal.pcbi.1002628 (PMC3415397; doi:10.1371/journal.pcbi.1002628)
Supplement: Text S4 — Complete source code for the PyDSTool package (version 0.88.120504). Includes API documentation and help files linking to web pages. This file is identical to the current public release on Sourceforge.net. (ZIP) [file pcbi.1002628.s004.zip › PyDSTool/html/PyDSTool.MProject.feature_node-class.html]

xml version="1.0" encoding="ascii"?


PyDSTool.MProject.feature\_node


| Home | Trees | Indices | Help | PyDSTool |
| --- | --- | --- | --- | --- |

Package PyDSTool ::
Module MProject ::
Class feature\_node

[hide private]

[frames] | no frames]

# Class feature\_node

source code

```
object --+    
         |    
   feature --+
             |
            feature_node
```

Known Subclasses:
:   - qt\_feature\_node
    - , ql\_feature\_node

---

Abstract super-class for feature regular nodes (supporting
sub-features).


|  |  |
| --- | --- |
| Instance Methods | [hide private] |

|  |  |
| --- | --- |
| \_\_init\_\_(self, name, description=`'``'`, pars=None, ref\_traj=None, subfeatures=None)  Sub-features is an ordered sequence of QL or QT feature instances which are (by default) evaluated in this order on a trajectory segment unless evaluation method is overridden. | source code |

|  |  |
| --- | --- |
| \_residual\_info(self, feats, sizes)  Update feats and sizes lists in place with metric info, if any. | source code |

|  |  |
| --- | --- |
| \_\_str\_\_(self)  str(x) | source code |

|  |  |
| --- | --- |
| \_\_repr\_\_(self)  str(x) | source code |

|  |  |
| --- | --- |
| \_\_getitem\_\_(self, featname)  Return named sub-feature | source code |

|  |  |
| --- | --- |
| propagate\_verbosity(self, sf) | source code |

|  |  |
| --- | --- |
| evaluate(self, target)  Default method: evaluate sub-features in order (assumes they are stored as a list). | source code |

|  |  |
| --- | --- |
| set\_ref\_traj(self, ref\_traj)  May or may not be used by the feature. | source code |

**Inherited from `feature`**:
`__call__`,
`__eq__`,
`__hash__`,
`__ne__`,
`finish`,
`info`,
`postprocess_ref_traj`,
`prepare`,
`reset_metric`,
`validate`

**Inherited from `feature`** (private):
`_find_idx`

**Inherited from `object`**:
`__delattr__`,
`__getattribute__`,
`__new__`,
`__reduce__`,
`__reduce_ex__`,
`__setattr__`


|  |  |
| --- | --- |
| Properties | [hide private] |

**Inherited from `object`**:
`__class__`


|  |  |
| --- | --- |
| Method Details | [hide private] |

|  |  |
| --- | --- |
| \_\_init\_\_(self, name, description=`'``'`, pars=None, ref\_traj=None, subfeatures=None)  *(Constructor)* | source code |

Sub-features is an ordered sequence of QL or QT feature instances
which are (by default) evaluated in this order on a trajectory segment
unless evaluation method is overridden.

For more sophisticated use of sub-features, they should be provided as
a dictionary mapping feature names to the feature instance.

Overrides: object.\_\_init\_\_

|  |  |
| --- | --- |
| \_\_str\_\_(self)  *(Informal representation operator)* | source code |

str(x)

Overrides: object.\_\_str\_\_: *(inherited documentation)*

|  |  |
| --- | --- |
| \_\_repr\_\_(self)  *(Representation operator)* | source code |

str(x)

Overrides: object.\_\_repr\_\_: *(inherited documentation)*

|  |  |
| --- | --- |
| evaluate(self, target) | source code |

Default method: evaluate sub-features in order (assumes they are
stored as a list).

Can override with more sophisticated method (e.g. for use with a
dictionary of sub-features).

Overrides: feature.evaluate

|  |  |
| --- | --- |
| set\_ref\_traj(self, ref\_traj) | source code |

May or may not be used by the feature. If not used, it will be
ignored.

Overrides: feature.set\_ref\_traj

  


| Home | Trees | Indices | Help | PyDSTool |
| --- | --- | --- | --- | --- |

|  |  |
| --- | --- |
| Generated by Epydoc 3.0.1 on Fri May 4 15:24:06 2012 | http://epydoc.sourceforge.net |
